# Connexin channels and hemichannels are modulated differently by charge reversal at residues forming the intracellular pocket

**DOI:** 10.1186/s40659-024-00501-5

**Published:** 2024-05-23

**Authors:** Felipe Villanelo, Peter J. Minogue, Jaime Maripillán, Mauricio Reyna-Jeldes, Joaquin Jensen-Flores, Isaac E. García, Eric C. Beyer, Tomás Pérez-Acle, Viviana M. Berthoud, Agustín D. Martínez

**Affiliations:** 1 Computational Biology Lab, Centro Basal Ciencia & Vida, Santiago, 8580702 Chile; 2 Facultad de Ingeniería, Arquitectura y Diseño, Universidad San Sebastián, Bellavista 7, Recoleta, Santiago, Chile; 3 Department of Pediatrics, University of Chicago, Chicago, IL 60637 USA; 4 Facultad de Ciencias, Centro Interdisciplinario de Neurociencias de Valparaíso, Instituto de Neurociencia, Universidad de Valparaíso, Valparaíso, Chile; 5 Laboratorio de Fisiología Molecular y Biofísica, Facultad de Odontología, Universidad de Valparaíso, Valparaíso, Chile; 6 Centro de Investigación en Ciencias Odontológicas y Médicas, Universidad de Valparaíso, Valparaíso, Chile

**Keywords:** Connexin, Hemichannel, Gap junction, Intracellular pocket

## Abstract

**Background:**

Members of the β-subfamily of connexins contain an intracellular pocket surrounded by amino acid residues from the four transmembrane helices. The presence of this pocket has not previously been investigated in members of the α-, γ-, δ-, and ε-subfamilies. We studied connexin50 (Cx50) as a representative of the α-subfamily, because its structure has been determined and mutations of Cx50 are among the most common genetic causes of congenital cataracts.

**Methods:**

To investigate the presence and function of the intracellular pocket in Cx50 we used molecular dynamics simulation, site-directed mutagenesis, gap junction tracer intercellular transfer, and hemichannel activity detected by electrophysiology and by permeation of charged molecules.

**Results:**

Employing molecular dynamics, we determined the presence of the intracellular pocket in Cx50 hemichannels and identified the amino acids participating in its formation. We utilized site-directed mutagenesis to alter a salt-bridge interaction that supports the intracellular pocket and occurs between two residues highly conserved in the connexin family, R33 and E162. Substitution of opposite charges at either position decreased formation of gap junctional plaques and cell–cell communication and modestly reduced hemichannel currents. Simultaneous charge reversal at these positions produced plaque-forming non-functional gap junction channels with highly active hemichannels.

**Conclusions:**

These results show that interactions within the intracellular pocket influence both gap junction channel and hemichannel functions. Disruption of these interactions may be responsible for diseases associated with mutations at these positions.

**Supplementary Information:**

The online version contains supplementary material available at 10.1186/s40659-024-00501-5.

## Background

The connexins (Cx) are a family of transmembrane proteins that form gap junctions containing channels that facilitate direct passage of ions and small molecules (≤ 1 kDa) between the cytoplasms of adjacent cells. Connexins also form “hemi-channels” that can open to connect the cytoplasm to the extracellular space. The general importance of connexin-mediated functions is demonstrated by the large variety of diseases caused by different connexin mutations, including deafness, neuropathies, cataracts, skin diseases, and oculodentodigital dysplasia (reviewed in [1, 2]). Many additional critical connexin functions have been identified through studies of gene ablations or other connexin mutations in mice (reviewed in [3]).

**Fig. 1:**
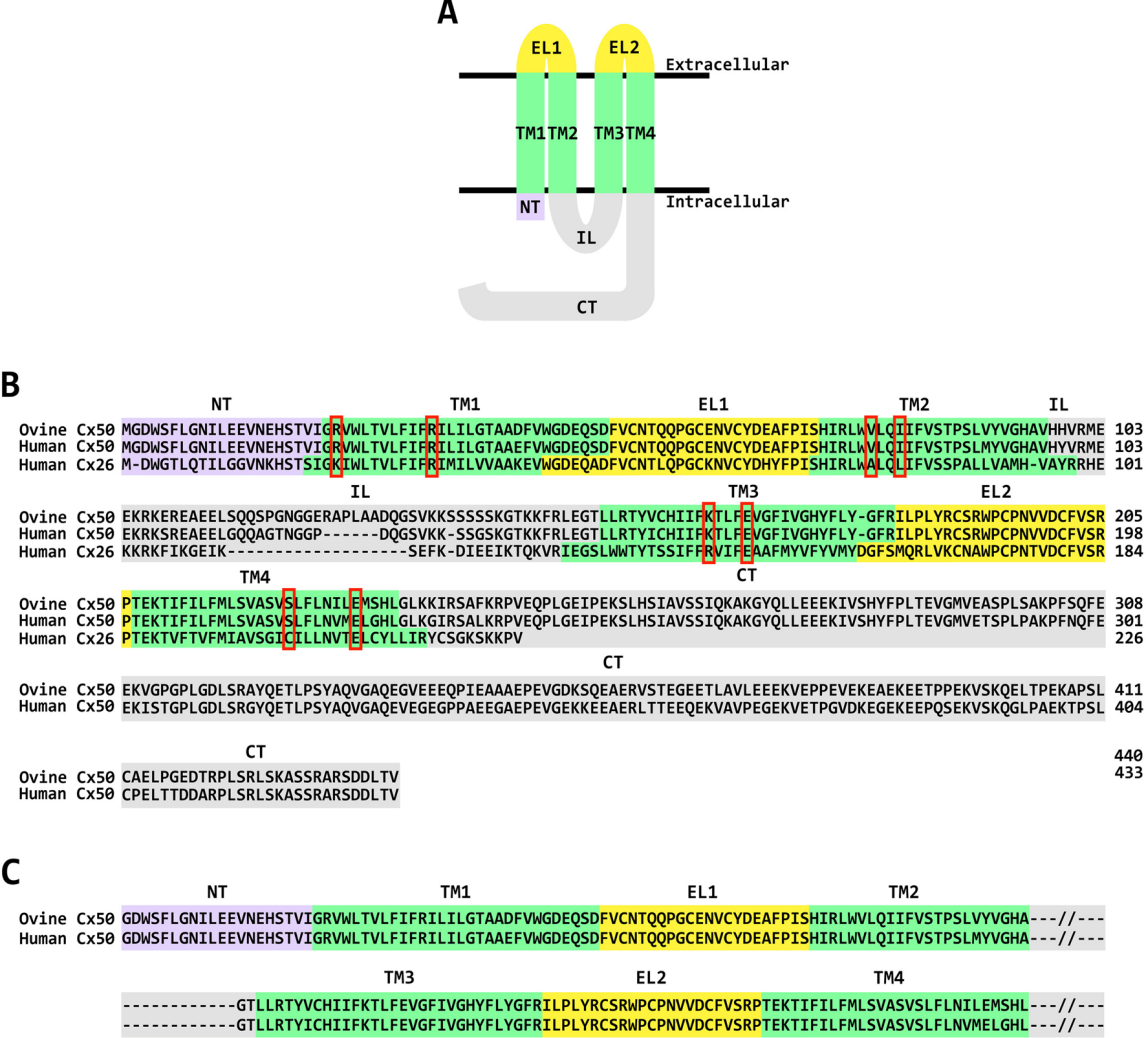
Connexin membrane topology and sequence conservation. **A** Diagram illustrating the membrane topology of a connexin monomer, indicating that it contains an amino terminus (NT, shown in mauve), four transmembrane domains (TM1–TM4, shown in green), two extracellular loops (EL1 and EL2, shown in yellow), an intracellular loop (IL, shown in gray), and a cytoplasmic carboxyl terminus (CT, shown in gray). **B** Sequence alignment of ovine and human Cx50 showing the high degree of amino acid sequence conservation between the two species, and of human Cx26. Amino acids have been assigned to specific protein domains in Cx50 according to the data reported in Fig. 4 by Myers et al. [6], and in Cx26 according to the data reported in Supplementary Fig. 3 by Maeda et al. [4]. The different domains have been enclosed by rectangles with colors that match those of the different domains in the diagram of the connexin monomer shown in **A**. The conserved amino acids involved in the formation of the intracellular pocket in Cx26 and Cx50 are indicated by the red boxes. **C** Sequence alignment of ovine and human Cx50 showing the amino acids included in our simulations based on the structure of the connexin channel reported by Myers et al. [6]. The intracellular loop and carboxyl terminus are not included, because they are not structured

Determination of detailed structures of connexin channels and hemichannels will help elucidate how connexin mutations contribute to disease. The polypeptide sequences of different connexins contain both similarities and differences. Each of the connexins has four transmembrane domains and two extracellular loops (Fig. 1A), containing many conserved amino acids. In contrast, the cytoplasmic intracellular loop and the carboxyl terminus differ among connexins.

Relatively high-resolution structures have been determined for hemichannels and gap junction channels composed of a few members of the connexin family, including Cx26 (the major connexin associated with deafness), Cx46 and Cx50 (two connexins associated with cataracts), and Cx31.3 (a connexin expressed mainly in oligodendrocytes that may only form hemichannels) [4–10]. The X-ray and cryo-electron microscopy studies have revealed the structure of the N-terminus and the transmembrane and extracellular domains; the cytoplasmic regions appear disordered (Fig. 1). These structures have been extended by modeling using molecular dynamics simulations [11–20].

While the structures of different connexins share many similarities, they have some differences. In the X-ray crystal structure of the human Cx26 gap junction, the hydrophobic amino acids of the N-terminal helix face the pore lumen, whereas in the cryo-electron microscopy structures of human Cx26 and the ovine Cx46/Cx50, these amino acids face the first transmembrane domain [4–7]. In the cryo-electron microscopy structure of Cx31.3 hemichannels, the N-terminal helix is oriented towards the cytoplasmic entrance to the pore, narrowing its diameter [8].

The intracellular pocket is a cavity near the N-terminal helix that was originally described in the wild-type human Cx26 hemichannel [11]. It is present in each of the six protomers, and it is lined by amino acids from different domains. The cavity is maintained by interactions between several of the amino acid side chains within the same protomer. The presence of the intracellular pocket has only been reported for Cx26 and Cx32, which belong to the β-connexin subfamily [11, 12]. However, many of the pocket-lining residues identified in Cx26 are conserved among members of the other subfamilies [11]. A variety of disease-linked connexin mutations have been identified at critical positions within this region (reviewed in [21]). Taken together, the amino acid conservation and the disease association of their replacement suggest that preservation of the intracellular pocket is important for proper channel function.

In the present study, we used molecular dynamics simulation to investigate the presence of the intracellular pocket in hemichannels formed by Cx50. We chose to study this connexin, since it is a member of the α-subfamily of connexins, and alterations of many different residues within Cx50 have been linked to disease. We identified the amino acid residues that may interact to form the Cx50 intracellular pocket and we studied the consequences of altering a supporting amino acid interaction that is conserved across connexin subfamilies. Our results suggest that interactions that form the intracellular pocket and the identities of the amino acid residues involved in these interactions are important for proper connexin targeting and gap junction and hemichannel functions.

## Results

### Simulation analysis shows the presence of an intramolecular and intracellular pocket in the wild-type human Cx50 hemichannel

To determine whether Cx50 hemichannels contain an intracellular pocket, we used molecular dynamics to model human Cx50 based on the cryo-electron microscopy structure of the ovine Cx50 hemichannel [7]. Ovine and human Cx50 share 96% amino acid identity and 100% similarity in the structured regions (Fig. 1B, C).

**Fig. 2:**
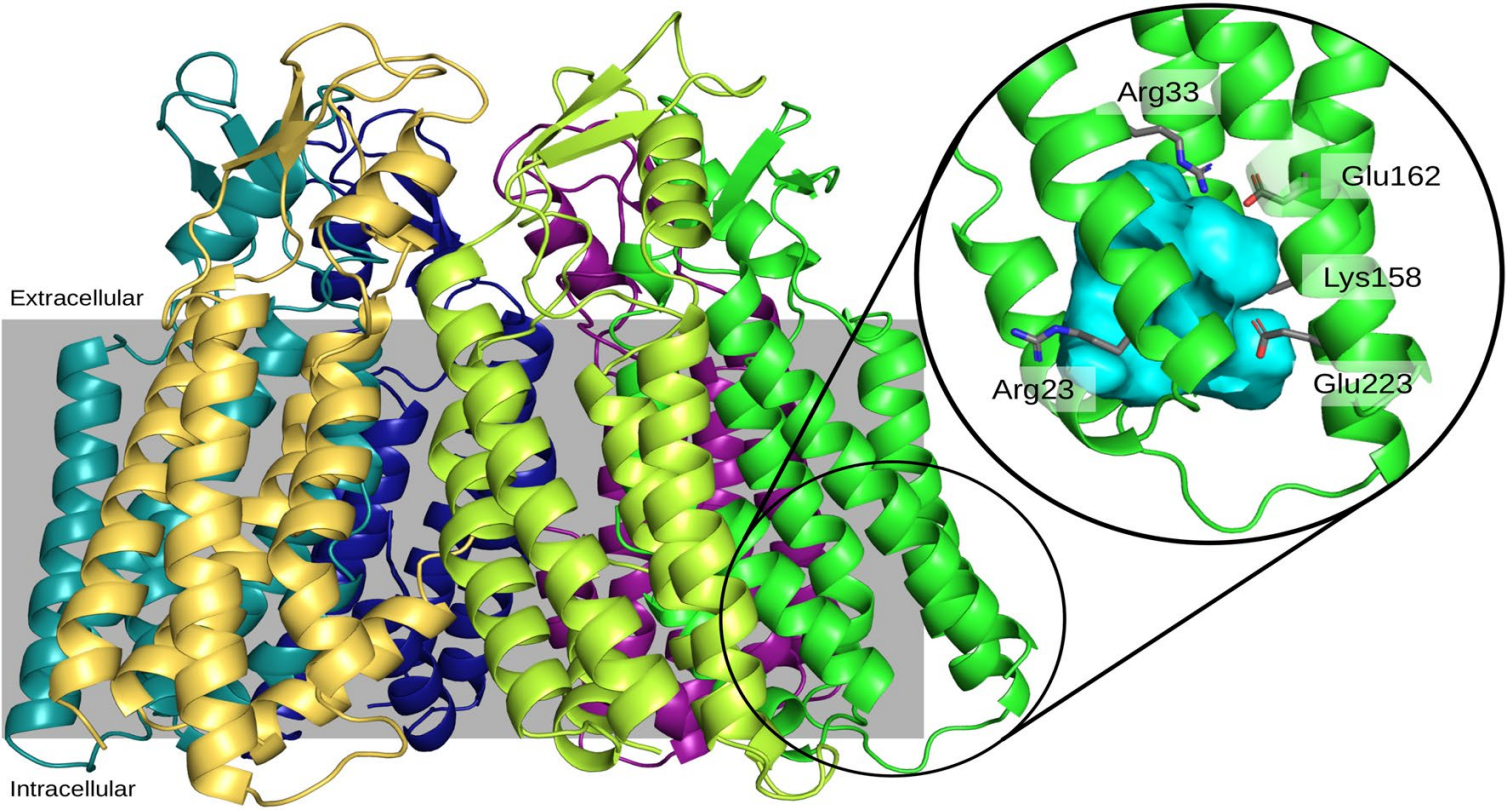
Molecular representation of a Cx50 hemichannel. Each protomer of the hexameric hemichannel is shown in ribbon representation in a different color. The position of the plasma membrane delimiting the extracellular and intracellular regions is represented in gray. The inset to the right highlights the location of the intracellular pocket in one of the protomers. Key residues are represented in sticks. The volume of the intracellular pocket that would be occupied by water molecules is shown in aqua

Analysis of the wild-type human Cx50 hemichannel structure confirmed the presence of an intracellular pocket (Fig. 2). Alignment of the Cx26 and Cx50 polypeptide sequences (Fig. 1) showed that several of the amino acid residues that form and line the intracellular pocket in human Cx26 are identical in Cx50, including R33 (R32 in Cx26), E162 (E147 in Cx26), and E223 (E209 in Cx26). Other pocket-lining amino acids are substituted by residues with similar properties, including (K22 in Cx26), V79 (A78 in Cx26), I82 (L81 in Cx26), K158 (R143 in Cx26), and S216 (C202 in Cx26). Therefore, we examined whether the salt-bridge interactions (K22-E209 and R32-E147) that support the pocket in the X-ray structure of the Cx26 protomers [11] also existed in Cx50 hemichannels. The expected corresponding interactions in human Cx50 would be between residues R23-E223 and R33-E162.

To assess whether similar interactions occur in the wild-type human Cx50 hemichannel, we used molecular dynamics simulations and determined whether the charged atoms on the side chains of these amino acids were at an interacting distance (≤ 4 Å) during the 100-ns simulation. The R33-E162 salt bridge is stable in all monomers in the wild-type Cx50 hemichannel. However, the R23-E223 salt bridge predicted from the X-ray structure of Cx26 hemichannels is not conserved in the cryo-electron microscopy structures of Cx50 or Cx26 intercellular channels [5, 7]. In the cryo-electron microscopy structures, R23 (located at the beginning of the first transmembrane domain) is not pointing toward E223, but instead points toward the pore lumen (Fig. 2). Because of its position and charge, K158 (the amino acid corresponding to R143 in human Cx26) forms a salt bridge with E223 in four of the six Cx50 monomers, and R23 does not interact with any nearby negatively charged residues, because their side chains are oriented away from R23. Therefore, we focused our attention on the R33-E162 interaction.

To determine whether alterations in the R33-E162 interaction affected the Cx50 hemichannel structure, we performed molecular dynamics simulations of Cx50 in which R33 or E162 was substituted by a charge reversal (Cx50R33E and Cx50E162R), by a neutralizing charge substitution (Cx50E162Q), and by a double opposite charge substitution (Cx50R33E,E162R). Similar to the wild-type Cx50 hemichannels, mutant Cx50 hemichannels reached a pseudo-equilibrium state during the 100-ns equilibration period. During the subsequent 100 ns of simulation, the root mean square deviation (RMSD) of wild-type Cx50, Cx50E162Q and Cx50R33E,E162R hemichannels remained under 2.0 Å, while the RMSD of the two single mutant (Cx50R33E and Cx50E162R) hemichannels was under 3.5 Å (Additional file 1: Fig. S1, left panel). The higher value of the RMSD in the single opposite charge substitution mutants may result from the instability introduced by the reversal of charge in the local environment of the R33-E162 salt bridge in the hemichannel structure of these mutants, which are not completely stabilized even after 200 ns (50-ns equilibration period + 50-ns equilibration period in the presence of 0.1 V/nm applied electrical field + 100-ns production simulation period). The RMSD of the Cx50R33E hemichannel is the highest, suggesting that this substitution is the most disruptive. Nevertheless, these RMSD values are of a similar magnitude to the reported resolution of the structure used as template, implying preservation of the overall structure of the channel. When the mutants were evaluated for structural movement at specific positions during the simulations, as informed by the root mean square fluctuation (RMSF), it became evident that Cx50R33E is the most flexible mutant. Overall, RMSF indicated that the extracellular loops were more flexible than the transmembrane domains as expected from the stabilizing effects of the lipid bilayer on them (Additional file 1: Fig. S1, right panel). Cx50R33E was more flexible than the other mutants in the extracellular loops. Surprisingly, all the mutant hemichannels were less flexible than wild type in the N-terminal helix region with Cx50R33E,E162R showing the highest difference compared to Cx50 (Additional file 1: Fig. S1, right panel).

**Fig. 3:**
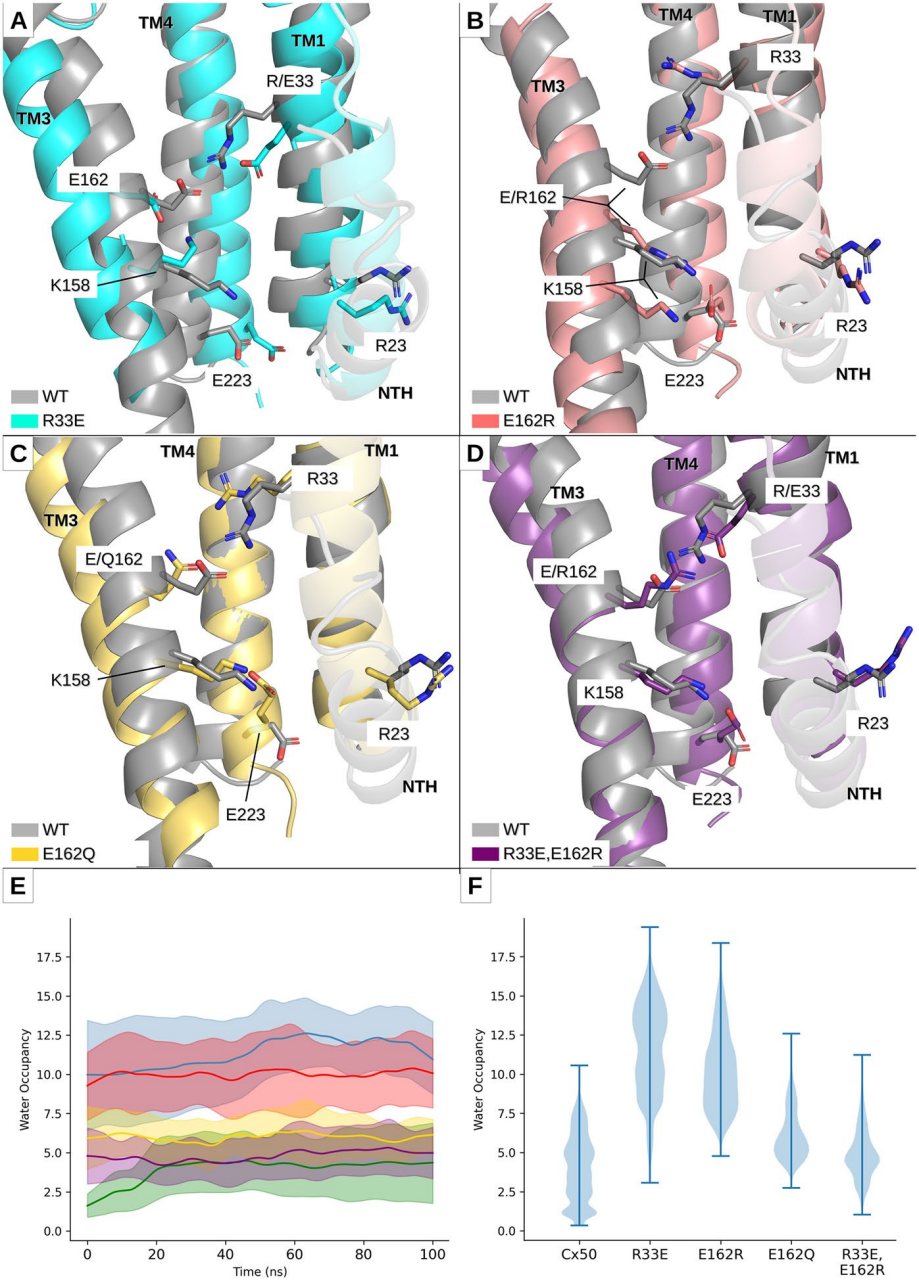
Comparison of the molecular environment around the intracellular pocket and of the distribution of water molecules between wild-type and mutant Cx50 protomers. **A**‒**D** The regions of the wild-type and mutant Cx50 protomers enclosing the majority of the volume of the intracellular pocket are shown in ribbon representation. The region of the wild-type protomer (shown in gray) has been superimposed upon the regions of the Cx50R33E (**A**), Cx50E162R (**B**), Cx50E162Q (**C**) and Cx50R33E,E162R (**D**) protomers to better illustrate the changes in tilting and twisting of their transmembrane domains compared with wild type. Simulations of wild-type Cx50 hemichannels show the salt-bridge interaction between amino acid residues R33 and E162 (R33-E162) within each protomer, and the absence of a salt-bridge interaction between amino acid residues R23 and E223. The ionic interaction between amino acid residues at positions 33 and 162 is lost in the single mutants, Cx50R33E, Cx50E162R and Cx50E162Q. In the Cx50R33E protomer, E162 can interact with K158 (**A**). In the wild-type Cx50, Cx50E162R, Cx50E162Q and Cx50R33E,E162R protomers, K158 can interact with E223 (**B**–**D**). This interaction does not occur in all six protomers, except in the case of the double charge substitution mutant hemichannels. In the protomer from the double substitution mutant, Cx50R33E,E162R, the mutated residues, E33 and R162, can form a salt-bridge interaction (**D**). All structures shown were extracted from the last frame of the corresponding simulation. **E** Graph shows the time-course of the number of water molecules inside the intracellular pocket during the 100-ns simulation in each system. Data are presented as the average (line) ± standard deviation (shade) of the 3 100-ns simulations performed for each system. **F** Violin plot shows the distribution of the number of water molecules inside the intracellular pocket. The data correspond to all frames from all three replicas for each system

**Fig. 4:**
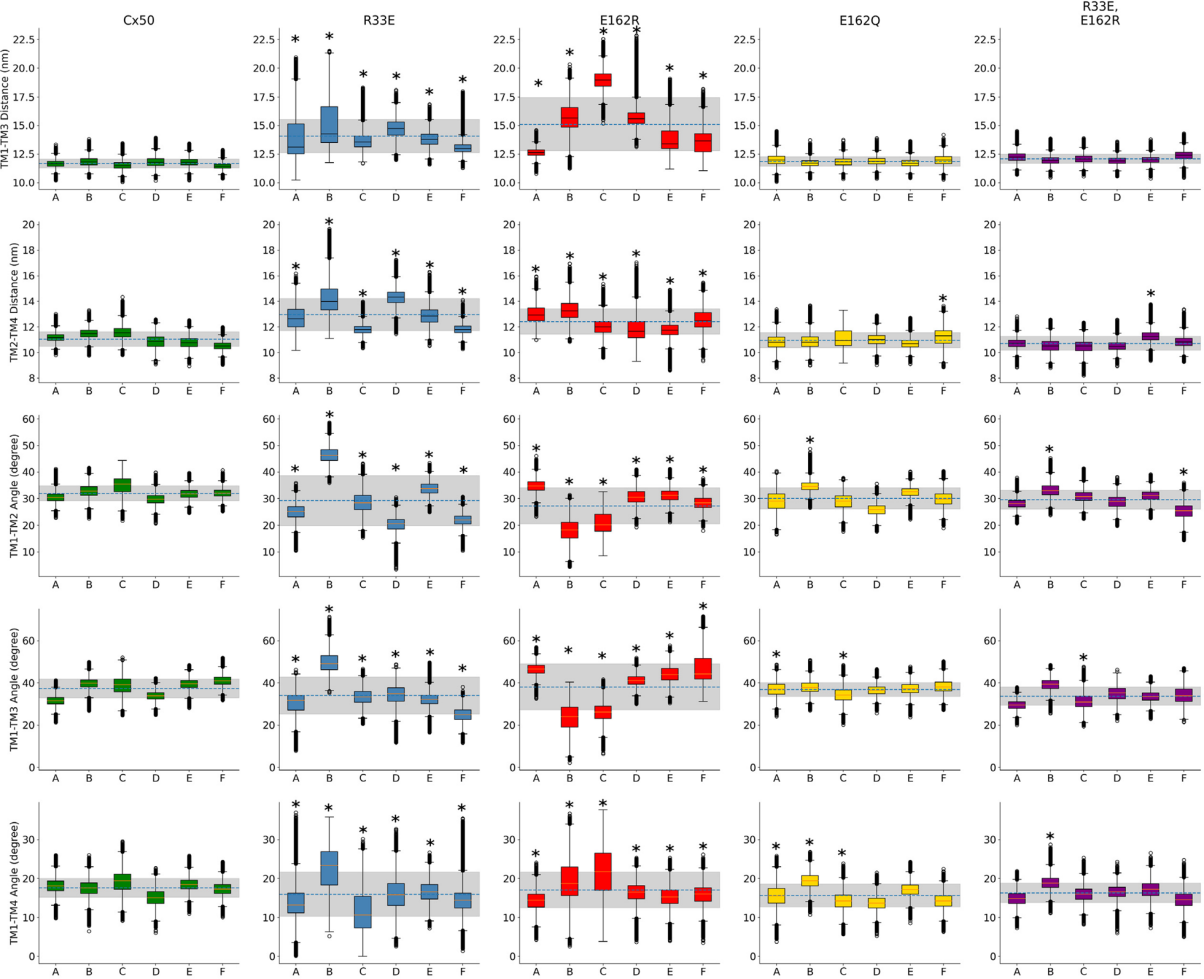
Distance and angle variations between the transmembrane domains of the mutant protomers compared with the wild-type Cx50 protomer. Graphs show the distance (top two rows) and the angle (bottom 3 rows) between transmembrane domains in Cx50, Cx50R33E, Cx50E162R, Cx50E162Q and Cx50R33E,E162R protomers. The distance between TM1 and TM3 was measured between amino acid residues 25 and 159, and that between TM2 and TM4 was measured between amino acid residues 85 and 220. The inclination of TM2, TM3 and TM4 with respect to TM1 was calculated by finding the best fitted straight line passing through the C_α_ of all amino acid residues in each transmembrane domain. The results are presented as box plots (mean ± S.D.) for each protomer (**A**‒**F**) using the measurement from all frames from all three replicas. The outliers are presented as empty circles. The horizontal dashed line corresponds to the average for all six protomers for each construct and the gray shade corresponds to the standard deviation. Asterisks indicate a significant difference vs. the corresponding monomer in wild-type Cx50 using Kruskal–Wallis test (p < 0.05)

Superposition of the intracellular pocket regions of each mutant upon the wild-type Cx50 structure revealed that the transmembrane domains of the different mutant protomers underwent slight tilts (with respect to the plane of the lipid bilayer) and rotations (Fig. 3). We quantified the changes in the orientation of the transmembrane domains by measuring the distance between transmembrane domains 1 and 3 (TM1 and TM3) and between transmembrane domains 2 and 4 (TM2 and TM4) for each protomer as the distance between the amino acid residues that are closest to the center of mass of each transmembrane domain. The TM1–TM3 and the TM2–TM4 distances were higher in the single opposite charge substitution mutants than in wild-type Cx50, whereas these distances were similar to wild type in the neutralizing charge substitution and double opposite charge substitution mutants (Fig. 4). When analyzed for each protomer, these distances were significantly different from wild type in every Cx50R33E and Cx50E162R protomer. In contrast, only the TM2–TM4 distance differed from wild type in the Cx50E162Q and Cx50R33E,E162R mutants, and it differed only in one of the six protomers. We also calculated the inclination plane of TM2, TM3 and TM4 with respect to TM1 by measuring the angle formed by each of these transmembrane domains relative to TM1. The average values of the angle between the different transmembrane domains and TM1 were within the same range for all constructs (Fig. 4, dashed line). However, the Cx50R33E and Cx50E162R mutants showed a much more pronounced dispersion in angle values between protomers than wild-type Cx50, Cx50E162Q or Cx50R33E,E162R as illustrated by the gray shading in Fig. 4, which represents the standard deviation. Similar to the analysis of the TM1–TM3 and TM2–TM4 distances, the TM1–TM2, TM1–TM3 and TM1–TM4 angles differed from wild type in every Cx50R33E and Cx50E162R protomer whereas these angles differed in 1–3 Cx50E162Q protomers and in 1–2 Cx50R33E,E162R protomers. These changes in tilting and twisting of the transmembrane domains would result in a large (Cx50R33E and Cx50E162R) or slight (Cx50E162Q) increase in the apparent volume of the intracellular pocket compared to wild type as predicted by the number of water molecules inside the intracellular pocket in the production simulations (Fig. 3E). The combined data from all protomers in the simulations, revealed that the volumes of the intracellular pockets in Cx50R33E and Cx50E162R hemichannels were on average greater (but more variable) than the volume of the wild-type hemichannels (Fig. 3F). Neutralization of the negative charge at position 162 (E to Q substitution) resulted in hemichannels with an intracellular pocket of slightly higher volume than wild-type hemichannels (Fig. 3F). In the Cx50R33E,E162R hemichannels, the pocket volume was less variable and was similar to that of wild-type hemichannels (Fig. 3F).

**Fig. 5:**
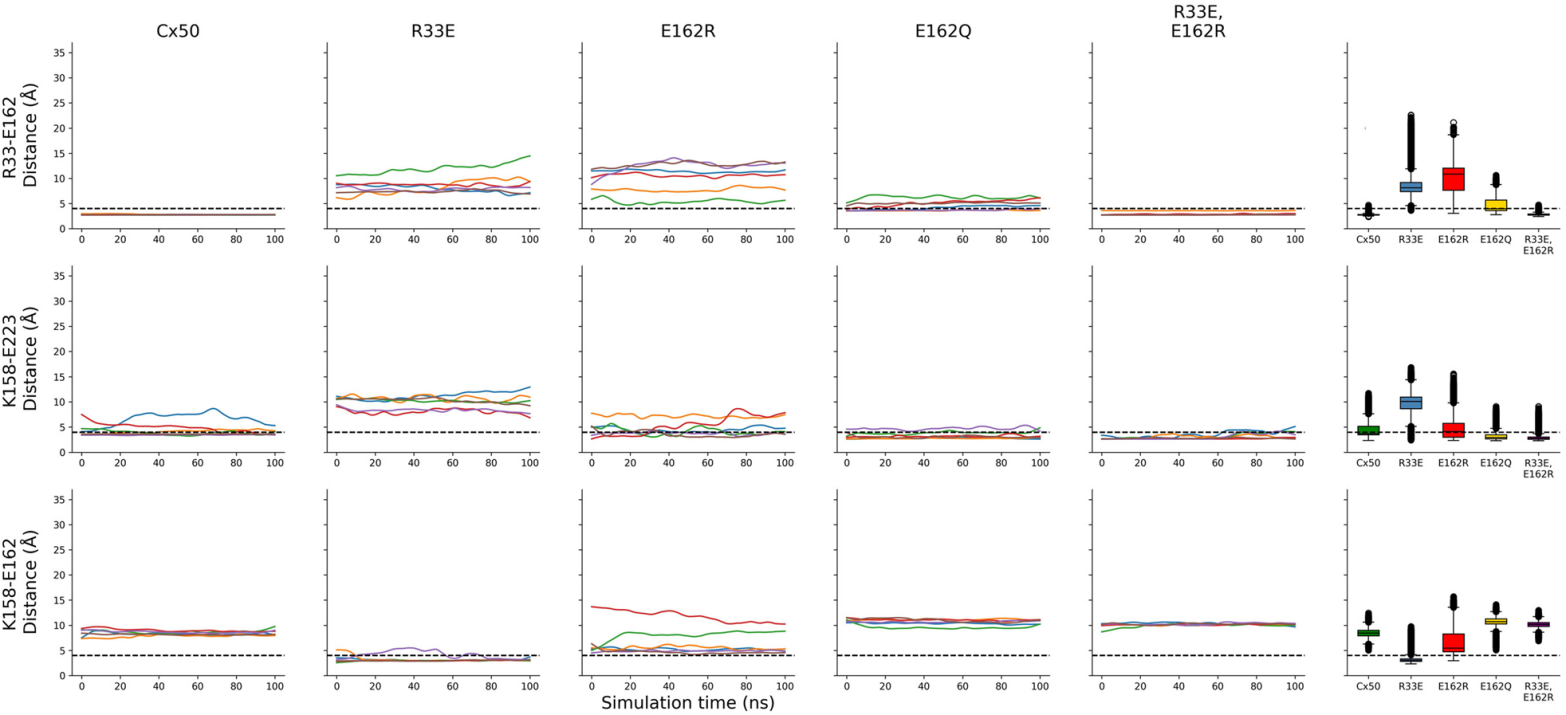
Amino acids within the intracellular pocket involved in salt-bridge formation. Graphs show the distance between selected pairs of amino acid residues: positions 33–162, positions 158-223, and positions 158-162 in wild-type and mutant Cx50. Distances were calculated from the center of mass of the terminal moieties of each residue (guanidinium for R; carboxylate for E; amide for Q). The graphs in the first five columns show the time-course of variation of the mean value of the distance in the three 100-ns simulation replicas for each protomer (plotted in a different color). The last column shows box plots of the same distances, with all protomers analyzed together. The black dashed line marks the 4 Å maximum distance limit above which two charged residues could not form a salt bridge

To test how mutations in the R33-E162 interaction affected interactions between the side chains of the other amino acids involved in formation of the intracellular pocket, we determined whether these amino acid residues were at an interacting distance during the 100-ns production simulation period for each protomer. As expected, the R33-E162 salt-bridge interaction was lost in the Cx50R33E, Cx50E162R and Cx50E162Q hemichannels, but this interaction was very stable in the double mutant hemichannel (similarly to wild type) (Fig. 5). As in the wild-type hemichannel, residue R23 never formed a salt bridge with E223 or interacted with the nearby negatively charged residues (E13 and E16 from the same protomer or E13 from the adjacent protomer) in the mutant Cx50 hemichannels. Interestingly, differences in interactions between the mutants and wild type were centered around the pore-lining residue K158. In wild-type Cx50, K158 forms a salt bridge with E223 in four of the six monomers, whereas this interaction is present in three of the six monomers in Cx50E162R hemichannels, in five of the six monomers in Cx50E162Q hemichannels, and in all six monomers in Cx50R33E,E162R hemichannels (Fig. 5). This structural change between wild-type and the double opposite charge substitution mutant hemichannels is the most relevant (i.e., the only structural change found) and appears to lock E223 in this interaction not allowing it to freely rotate as in the other structures. In contrast, K158 forms a salt bridge with E162 in Cx50R33E hemichannels in 5 of the 6 protomers (Fig. 5).

### Disruption of the R33-E162 interaction affects gap junctional plaque formation and function

**Fig. 6:**
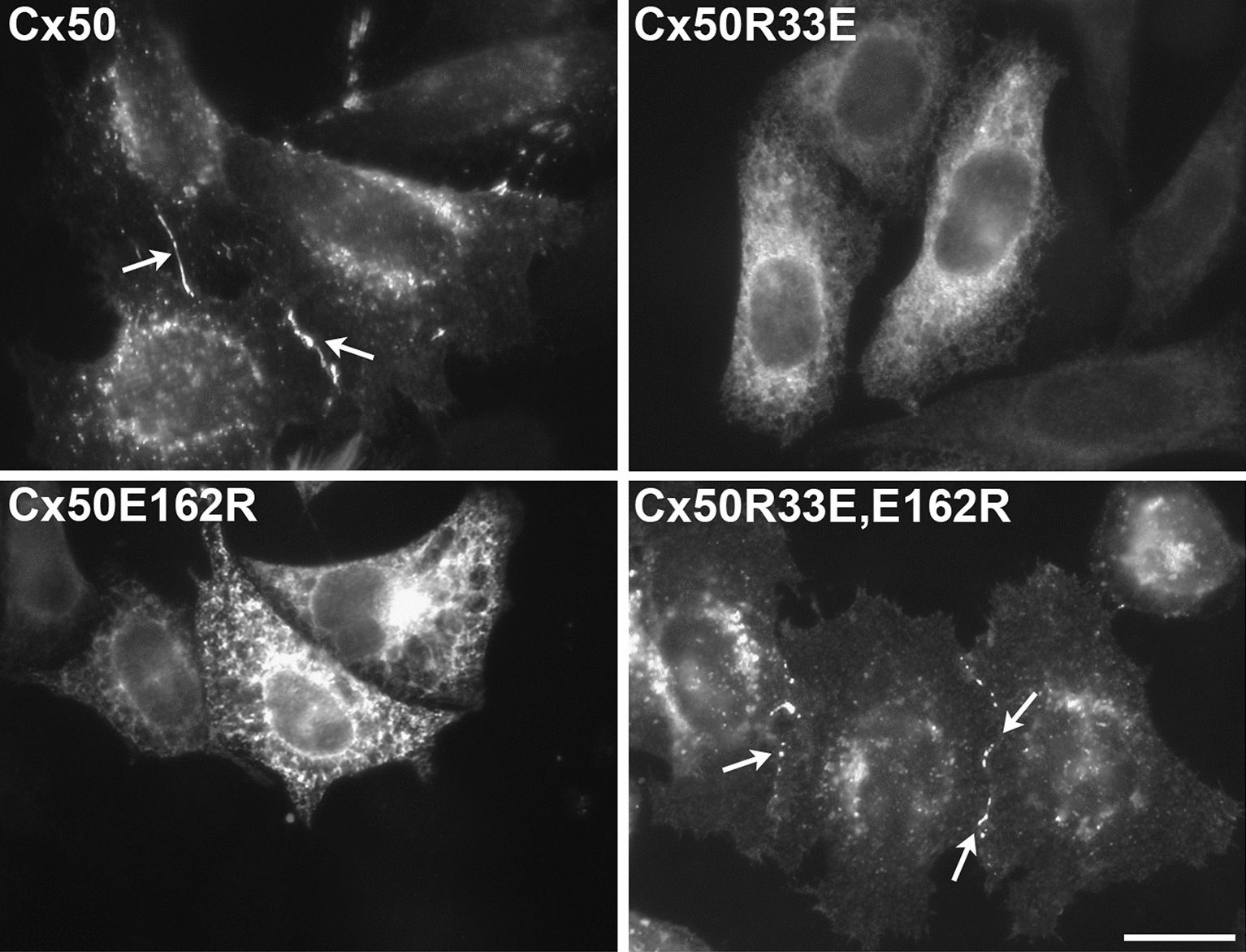
Elimination of the R33-E162 interaction in the intracellular pocket impairs gap junctional plaque formation. Photomicrographs show the distribution of immunoreactive Cx50, Cx50R33E, Cx50E162R and Cx50R33E,E162R in transfected HeLa cells. Gap junctional plaques formed by Cx50 and the double mutant, Cx50R33E,E162R, are indicated by arrows. Scale bar, 20 μm

To determine the cellular effect(s) of alteration of the R33-E162 interaction, we expressed the single and double opposite charge substitution mutants of Cx50 in HeLa cells and studied their cellular distribution by immunofluorescence. Wild-type Cx50 localized to gap junctional plaques as expected (Fig. 6). In contrast, the single mutants, Cx50R33E and Cx50E162R, predominantly showed a cytoplasmic distribution (Fig. 6) with very rare formation of gap junctional plaques of low fluorescence intensity (not shown). In contrast, the double mutant, Cx50R33E,E162R, formed abundant gap junctional plaques (Fig. 6).

**Fig. 7:**
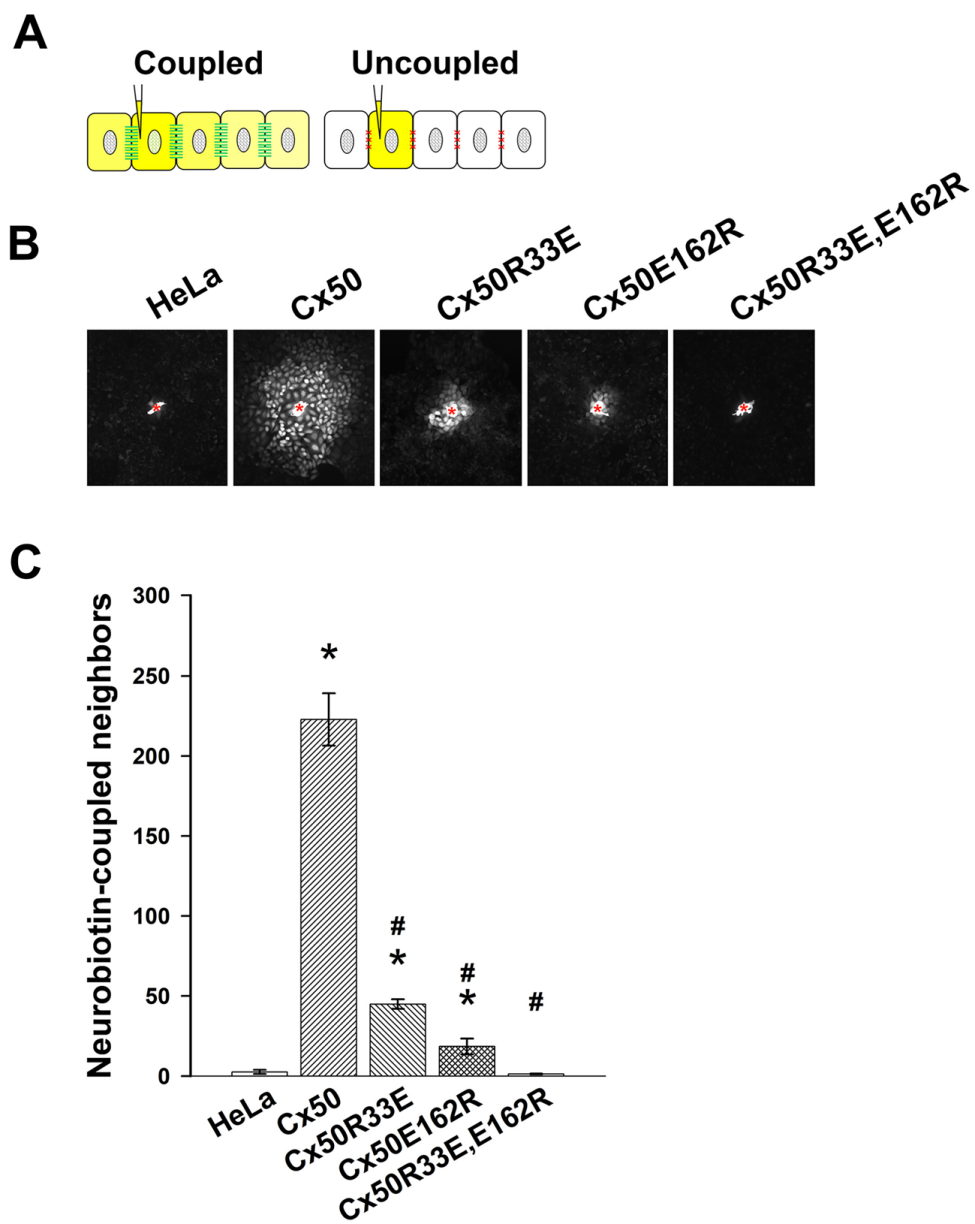
Charge reversal at R33 or E162 reduces intercellular transfer of Neurobiotin. **A** Left, schematic diagram illustrating the intercellular transfer of a gap junction tracer (shown in yellow) from a microinjected cell to its neighboring coupled cells when gap junction channels are open (paired green lines). Right, schematic diagram illustrating the lack of intercellular transfer of a gap junction tracer (shown in yellow) from a microinjected cell to its neighboring cells when cells are uncoupled (red Xs in between the cells). **B** Photomicrographs show representative examples of intercellular transfer of Neurobiotin in HeLa, HeLa-Cx50, HeLa-Cx50R33E, HeLa-Cx50E162R and HeLa-Cx50R33E,E162R cells after microinjection of the tracer into one cell of the cluster (indicated by the red asterisk). **C** Bar graph shows the quantification of intercellular transfer of Neurobiotin in untransfected HeLa cells (HeLa) and HeLa cells stably transfected with Cx50, Cx50R33E, Cx50E162R or Cx50R33E,E162R. The results are presented as mean (bar) ± S.E.M. (error bars). A minimum of ten independent microinjections were analyzed (n = 15 for HeLa, 24 for Cx50, 13 for Cx50R33E, and 10 for Cx50E162R and Cx50R33E,E162R). Asterisks indicate significant differences vs. HeLa cells (p < 0.012); ^#^indicates significant differences vs. HeLa-Cx50 cells (p < 10^–9^)

To test the ability of the mutants to support intercellular communication, we examined intercellular transfer of Neurobiotin (a gap junction tracer; charge: + 1; MW: 322.8) in stably transfected HeLa cells. For this purpose, we microinjected Neurobiotin into one cell of a cluster and determined the number of cells to which the tracer transferred. If cells are coupled, the tracer transfers to neighboring cells (which can be quantified by counting tracer-containing cells); if cells are uncoupled, the tracer remains in the microinjected cell (as shown diagrammatically in Fig. 7A). In untransfected HeLa cells, Neurobiotin transferred to very few or no neighbors (Fig. 7B, C); on average, it transferred to 2.6 ± 1.2 neighboring cells. As expected, wild-type Cx50 supported extensive intercellular communication; Neurobiotin transferred to 222.8 ± 16.4 neighboring cells in HeLa-Cx50 cells (Fig. 7B, C). Consistent with the paucity of gap junctional plaques in cells expressing the single mutants, they only supported low levels of intercellular coupling. Neurobiotin transferred to 45 ± 2.9 cells (Cx50R33E) and 18.5 ± 4.9 cells (Cx50E162R) (Fig. 7B, C). These values are significantly lower compared with HeLa-Cx50 cells, but significantly higher than those determined in untransfected cells (Fig. 7C). Surprisingly, cells expressing the double mutant transferred Neurobiotin to only 1.3 ± 0.3 neighboring cells, a value not significantly different from untransfected cells (Fig. 7B, C), indicating that the gap junctional plaques formed by this mutant contain non-functional channels.

### The Cx50 double mutant, Cx50R33E,E162R, shows gain-of-hemichannel function

**Fig. 8:**
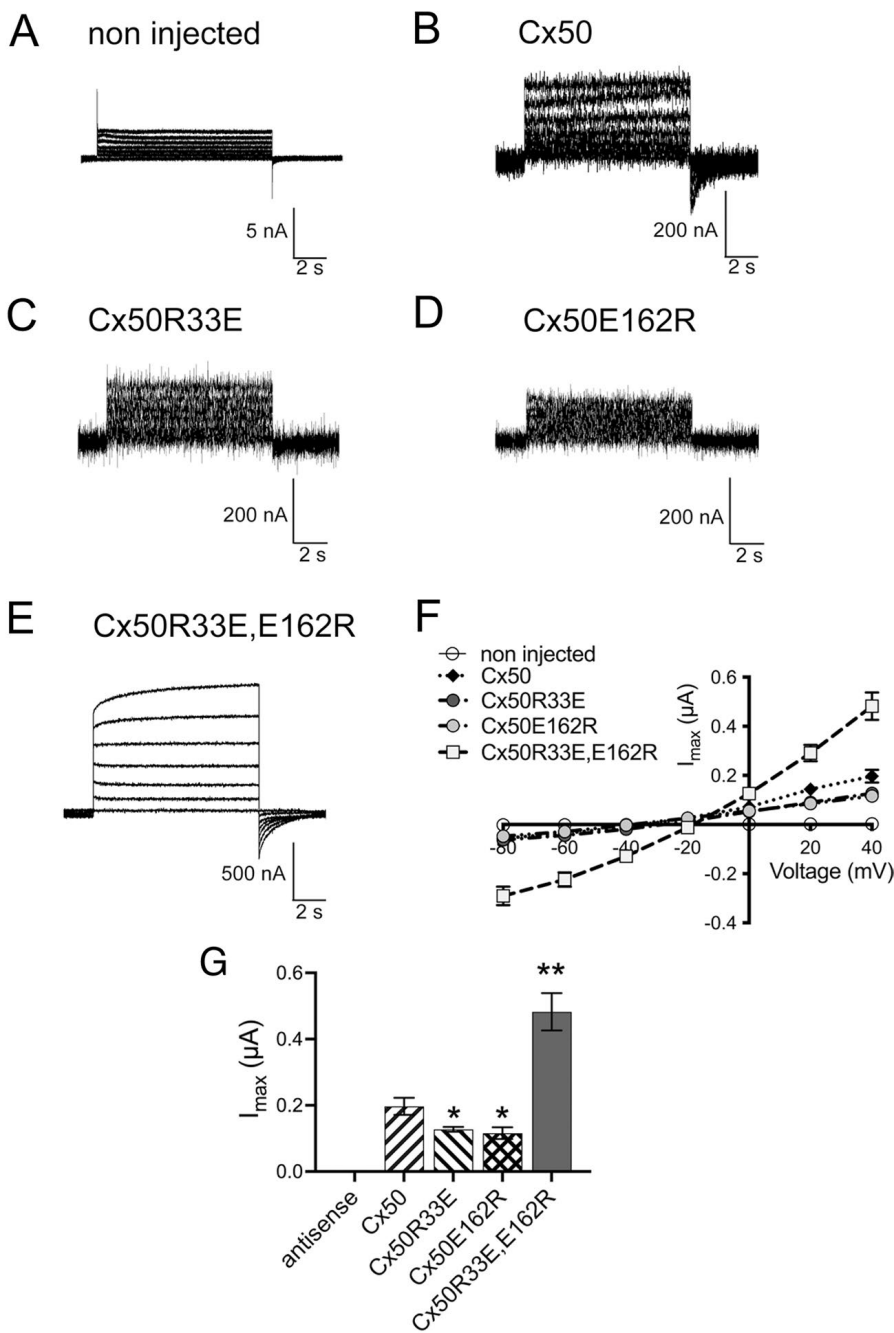
The Cx50 double mutant, Cx50R33E,E162R, induces gain of hemichannel function. Membrane currents were elicited and recorded from oocytes under two-electrode voltage clamp in response to 10-s depolarizing voltage steps from − 80 mV to + 40 mV in 20-mV increments from a holding potential of − 80 mV. **A**‒**E** Representative families of macroscopic current traces recorded from oocytes injected with no cRNA (**A**), or with equal amounts of wild-type Cx50 (**B**), Cx50R33E (**C**), Cx50E162R (**D**) or Cx50R33E,E162R (**E**) cRNAs. **F** Graph shows the steady-state current–voltage relationships of the macroscopic hemichannel tail currents for wild-type and mutant Cx50 obtained after returning the voltage from each depolarizing step to the holding potential of − 80 mV. **G** Graph shows the instantaneous tail currents (I_max_) for wild-type and mutant Cx50 hemichannels obtained after the + 40 mV depolarizing step. Data are presented as mean ± S.E.M. (n = 10 oocytes for each construct). *p < 0.05 and **p < 0.01 indicate significant differences vs. Cx50 cRNA-injected oocytes. Oocytes were obtained from at least three frog donors

Because connexins can also form voltage-dependent functional hemichannels, we tested whether Cx50R33E, Cx50E162R, or Cx50R33E,E162R differed in their ability to form active hemichannels compared with wild-type Cx50. We determined macroscopic hemichannel currents as a function of voltage in *Xenopus laevis* oocytes microinjected with antisense Cx38 oligonucleotides and no connexin cRNA or the respective Cx50 cRNAs. Representative examples of hemichannel current recordings are shown in Fig. 8A–E. Oocytes expressing wild-type Cx50 developed macroscopic currents after application of depolarizing voltage steps in the presence of 1.8 mM extracellular Ca^2+^ (Fig. 8B, F), which is consistent with previous reports [22, 23]. Negligible hemichannel currents were recorded from oocytes expressing Cx50R33E or Cx50E162R, even after application of large depolarizing voltages (Fig. 8C, D, F). In contrast, oocytes expressing the double mutant, Cx50R33E,E162R, showed large macroscopic currents throughout the entire range of voltages tested (Fig. 8E, F). The amplitude of the maximal current generated by the double mutant at + 40 mV (I_max_) was on average 2.4-times as great as the value recorded from oocytes expressing wild-type Cx50 (Fig. 8G; p < 0.01), whereas those of the single mutants were smaller than wild type (Fig. 8G; p < 0.05).

### The Cx50 double mutant, Cx50R33E,E162R, supported increased ethidium uptake and ATP release

**Fig. 9:**
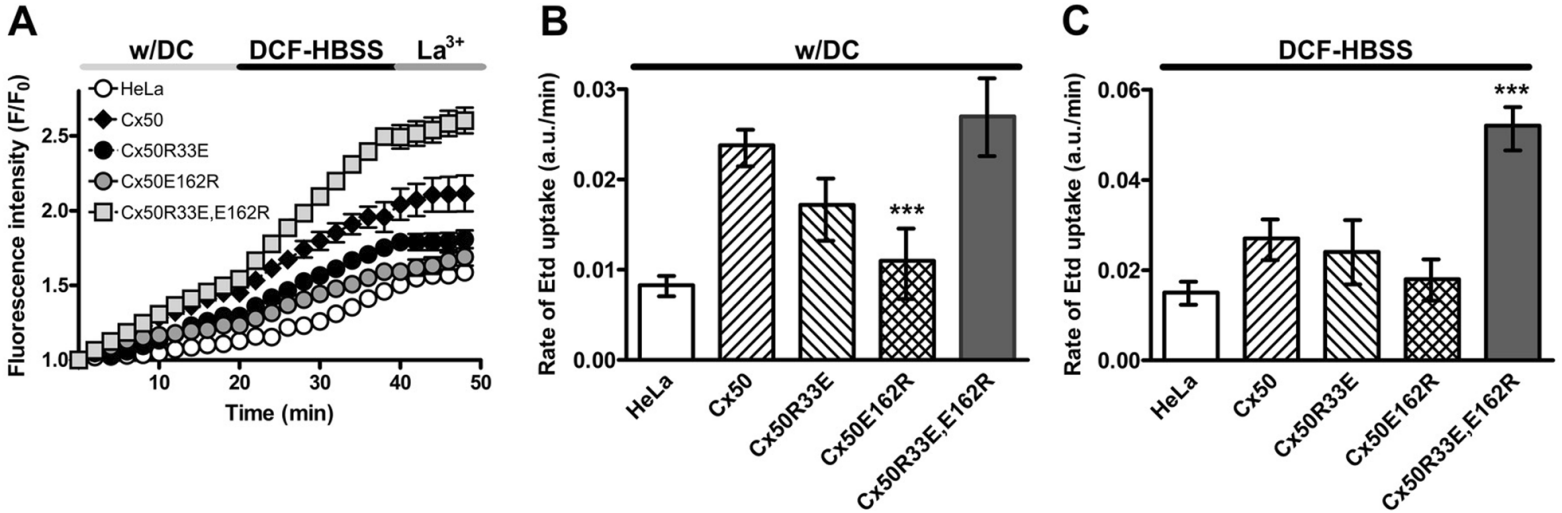
Cx50R33E,E162R increases ethidium uptake in divalent cation-free solution. **A** Graph shows the increase in cellular fluorescence intensity in arbitrary units (F/F_0_) after ethidium uptake when cells were incubated under physiological extracellular divalent cation concentrations (w/DC) for 20 min, followed by 20 min in Ca^2+^- and Mg^2+^-free Hanks’ balanced salt solution (DCF-HBSS), and, subsequently, in DCF-HBSS in the presence of 100 μM La^3+^ to block connexin hemichannels (La^3+^). **B**, **C** Bar graphs show the rate of ethidium uptake determined by calculating the slope of ethidium uptake in arbitrary units/minute (a.u./min) from the data presented in **A** under physiological conditions (**B**) or in divalent cation-free solution (**C**). Data are presented as mean ± S.E.M. (n = 3). ***p < 0.001 indicates significant differences vs. HeLa-Cx50 cells

We also considered that the R33-E162 interaction mutants might also affect hemichannel passage of molecules. We determined the ability of expressed connexin hemichannels to facilitate uptake of ethidium, a positively charged molecule (charge: + 1; MW: 394.5), in solutions containing or lacking extracellular divalent cations (in the absence or presence of 100 μM La^3+^, a non-selective connexin hemichannel blocker). Untransfected HeLa cells showed very low levels of ethidium uptake independent of the experimental condition (Fig. 9), consistent with previous reports on parental HeLa cells [24, 25]. In the presence of divalent cations, HeLa-Cx50R33E and HeLa-Cx50R33E,E162R cells showed a similar rate of dye uptake to HeLa-Cx50 cells (Fig. 9B). In contrast, the rate of ethidium uptake in HeLa-Cx50E162R was similar to that of untransfected cells and lower than that in cells expressing wild-type Cx50 (Fig. 9A, B). In the absence of divalent cations, the rate of ethidium uptake in cells expressing Cx50R33E or Cx50E162R was similar to that in HeLa-Cx50 cells and untransfected HeLa cells (Fig. 9A, C). Remarkably, cells expressing Cx50R33E,E162R showed a much greater rate of ethidium uptake than HeLa-Cx50 in divalent cation-free solution (Fig. 9A, C; p < 0.001). These results are consistent with the electrophysiological experiments indicating that the double mutant Cx50R33E,E162R forms hemichannels with increased activity.

**Fig. 10:**
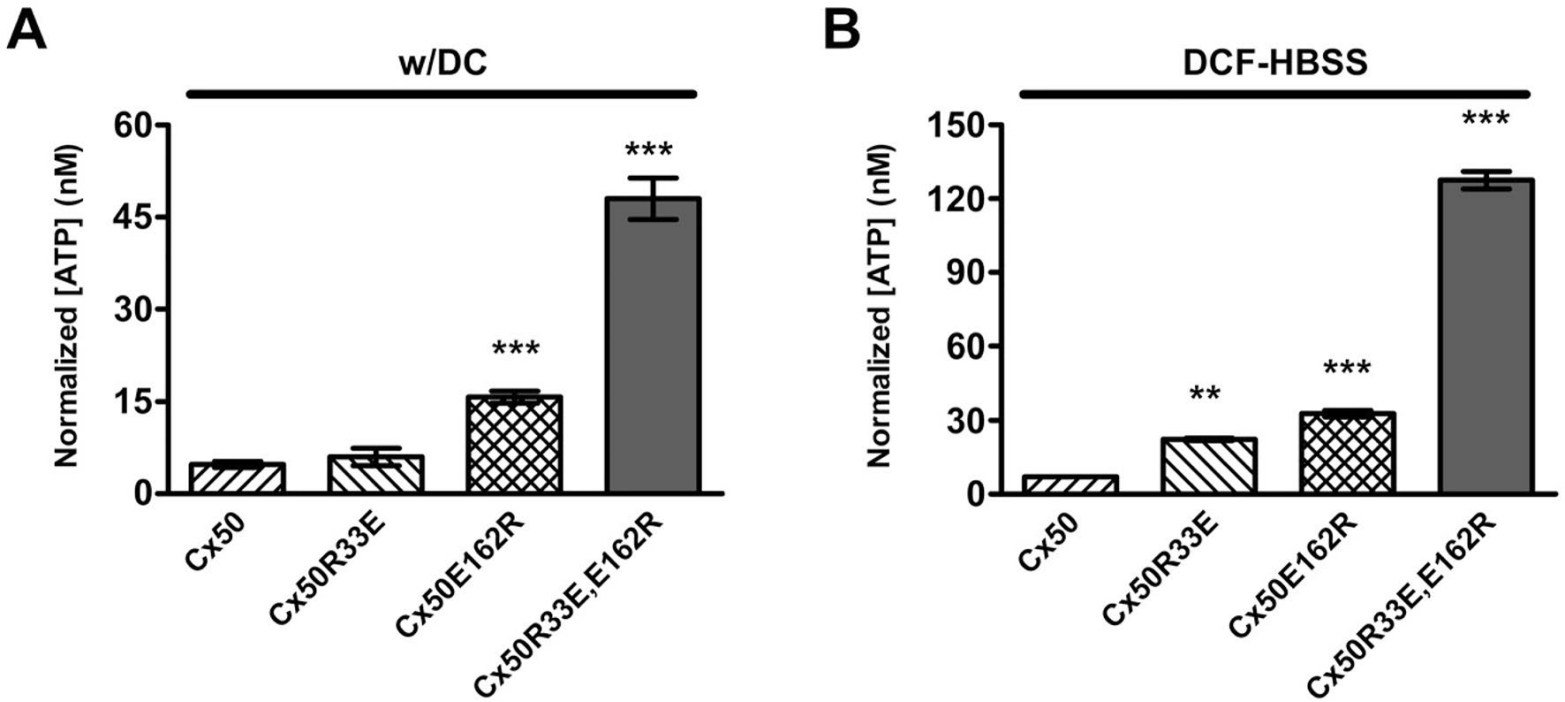
The Cx50 single and double mutants containing an arginine at position 162 increase ATP release. **A** Graph shows the normalized concentration of ATP released from HeLa cells stably transfected with Cx50, Cx50R33E, Cx50E162R or Cx50R33E,E162R under physiological extracellular divalent cation concentrations (w/DC). **B** Graph shows the normalized concentration of ATP released from HeLa cells stably transfected with Cx50, Cx50R33E, Cx50E162R or Cx50R33E,E162R after a 30-min incubation in Ca^2+^- and Mg^2+^-free Hanks’ balanced salt solution (DCF-HBSS). Data are presented as mean ± S.E.M. (n = 4). **p < 0.01 and ***p < 0.001 indicate significant differences vs. HeLa-Cx50 cells

To test the possibility that the R33-E162 substitution mutants also affected hemichannel function for negatively charged molecules, we measured the release of intracellular ATP (charge: − 3; MW: 507.18), since previous studies have shown that hemichannel hyperactivity is associated with increased ATP release [26]. In the presence of extracellular divalent cations, cells expressing Cx50 or Cx50R33E showed low levels of ATP release. In contrast, cells expressing Cx50E162R or Cx50R33E,E162R released 3 or 9 times (respectively) the amount of ATP released by cells expressing wild-type Cx50 (Fig. 10A; p < 0.001). In divalent cation-free solution, cells expressing each of the Cx50 mutants released more ATP than HeLa-Cx50 cells although to different extents (Fig. 10B). Relative to HeLa-Cx50 cells, ATP release was 3.3 times as great for HeLa-Cx50R33E cells, 4.8 times as great for HeLa-Cx50E162R cells, and 19 times as great for the double mutant (p < 0.01 for Cx50R33E and p < 0.001 for Cx50E162R and Cx50R33E,E162E). These results provide additional confirmation that the double mutant forms hyperactive hemichannels.

## Discussion

Our molecular dynamics modeling demonstrates that human Cx50 contains an intramolecular intracellular pocket that shares similarities with the intracellular pocket described in Cx26 [11]. One of the interactions that contributes to the pocket formation in Cx26 is conserved by the corresponding amino acids in Cx50: the salt-bridge interaction between R33 and E162 is present in all the Cx26 and Cx50 structures [4–7].

The R33 and E162 salt-bridge interaction that participates in the formation of the intracellular pocket occurs between two amino acid residues that are highly conserved among the members of the connexin family. Among the expressed human connexins, R33 is absolutely conserved. The position corresponding to E162 contains glutamate in all but 2 of the 20 connexins (where it is replaced by aspartate) [27]. This conservation suggests the importance of these residues for the normal behavior of connexins. Our results show that disruption of this interaction by reversal of a single charge at these positions in Cx50 decreased intercellular transfer of Neurobiotin, consistent with the near absence of gap junctional plaque formation. Re-formation of a salt bridge between amino acid residues at these positions by double charge reversal substitution (Cx50R33E,E162R) recovered the formation of gap junctional plaques similar to wild-type Cx50, but it did not restore gap junctional function. These data imply that formation of gap junctional plaques requires a salt bridge between the amino acid residues at these positions; however, the charges at the individual positions (or the amino acid identities) are critical for normal gap junctional channel function.

The almost complete lack of gap junctional plaque formation by the single charge substitution mutants, Cx50R33E and Cx50E162R, may reflect an inability of hemichannels to traffic to the plasma membrane or dock or cluster due to the increased TM1–TM3 and TM2–TM4 distances and higher protomeric variation in the inclination of the different transmembrane domains with respect to TM1. Because most of the protein localized in the cytoplasm, it is likely that these structural alterations impaired trafficking. However, since these mutants supported some gap junctional function and ATP release, it is likely that the transmembrane domain movements prevented hemichannels within the plasma membrane from docking and/or clustering to form gap junction plaques. This interpretation is supported by the observation that the double substitution mutant formed gap junctional plaques; this mutant has similar TM1–TM3 and TM2–TM4 distances to wild-type Cx50, low dispersion in the angular inclination of the transmembrane domains and an overall apparent number of water molecules in the intracellular pocket similar to wild-type Cx50.

Opening of wild-type Cx50 hemichannels allowed the passage of ethidium and ATP similarly to hemichannels formed by other connexins, including Cx26, Cx32 and Cx43 [24, 25, 28–31]. Permeation of ATP through connexin hemichannels has been directly demonstrated for Cx43 [32]. Unlike hemichannels formed by Cx26 or Cx50 with a hemagglutinin epitope tag at its N-terminus [25, 33], the hemichannel activity of wild-type Cx50 was similar in divalent cation-containing and divalent cation-free solutions. The different behavior of Cx50 hemichannels in this study compared with that by Zhang et al. [33] likely results from the addition of an epitope tag to the N-terminus in the report by Zhang et al., as implied by the altered channel activity and properties caused by appending sequences to the N-terminus (and C-terminus) of Cx43 [24, 34]. Indeed, the N-terminal domain of connexins plays a major role in hemichannel and gap junctional channel voltage gating and permeability [13, 35–39].

Decreased gap junction channel function with increased hemichannel activity has been reported in several cases [26, 40–49] and was observed for some of the Cx50 mutants analyzed in this study. Alteration of the R33-E162 salt-bridge interaction affected the behavior of Cx50 hemichannels. The effect and its magnitude depended on the charge of the permeating species and the concentration of Ca^2+^ in the bathing solution. The most interesting results were obtained with the double charge substitution mutant, Cx50R33E,E162R. This mutant showed increased hemichannel currents, increased ethidium uptake in divalent cation-free solution, and increased release of ATP in the presence and in the absence of extracellular divalent cations. In contrast, the single opposite charge substitution mutant hemichannels supported increased release of ATP in the absence of divalent cations, but they did not support currents (in the presence of divalent cations) or ethidium uptake above wild-type Cx50 levels (independent of the extracellular concentration of divalent cations). Because Cx50E162R hemichannels supported increased ATP release even in the presence of divalent cations, this suggests that single opposite charge substitution at position 162 is sufficient to increase release of ATP through Cx50 hemichannels.

Several mechanisms likely contributed to the functional alterations in the opposite charge substitution mutants. Among the structural changes, alterations in the interactions of K158 and tilting and rotation of the transmembrane domains in the Cx50 mutant hemichannels are likely the most relevant. K158 (in Cx50) corresponds to R143 in Cx26; this residue has been shown to play an essential role in Cx26 hemichannel permeability [11]. Thus, changing the number of protomers within a hemichannel in which K158 interacts with E223 or causing a new interaction with E162 instead (as it occurs in Cx50R33E hemichannels) appears to be crucial for formation of active hemichannels. In addition, locking E223 in all protomers and impeding its free rotation likely contributed to the lack of formation of functional gap junctional channels in Cx50R33E,E162R. It might have been interesting to look for this interaction in simulations based on the closed Cx50 channel to eliminate any potential bias towards an open conformation in our simulations. Unfortunately, this closed structure has not been determined. Structural changes in the transmembrane domains of the single opposite charge substitution mutants may have also contributed to the formation of functional hemichannels by the single opposite charge substitution mutants.

All Cx50 mutant hemichannels showed a decrease in flexibility of the N-terminal helix that may have impaired hemichannel and gap junctional channel regulation since movement of the N-terminus has been implicated in gating [13, 35–39]. This change in N-terminal helix flexibility may have played a more significant role for the behavior of the Cx50R33E,E162R mutant, since this mutant showed the largest difference compared to wild type.

Taken together, the results presented here show that the identities of the amino acid residues at positions 33 and 162 (and their charges) have critical roles in forming gap junctional channels that support intercellular communication and in minimizing Cx50 hemichannel activity under physiological conditions. Because R33 and E162 are highly conserved across connexins, these results can explain why mutations at these positions (and the corresponding positions in other connexins) lead to disease. Cx26E147K has been linked to autosomal recessive sensorineuronal deafness [50]. Cx32R32K and Cx32E146K have been linked to X-linked Charcot-Marie Tooth disease type 1 [51]. Cx46R33L and Cx50E162K have been linked to autosomal dominant cataracts [52, 53]. Since the structure of the Cx46 and Cx50 hemichannels are highly similar [6, 7], our current results suggest that either of these mutations would cause a significant decrease in gap junctional coupling between lens fiber cells, which would impair the circulation of ions and fluid through the organ and alter homeostasis [54, 55]. In addition, the E162K substitution in Cx50 would increase release of ATP, even in the presence of physiological concentrations of extracellular Ca^2+^, which may lead to its intracellular depletion. The alteration of the lens circulation, lens cell homeostasis and depletion of ATP (in the case of Cx50E162K) would favor the formation of cataracts.

## Conclusions

We have shown that human Cx50 contains an intracellular pocket. By altering a conserved interaction that stabilizes it, we have shown that this pocket is important for connexin folding, trafficking, and modulation of hemichannel and gap junction channel function. These results provide a mechanistic explanation by which mutants of amino acid residues forming the intracellular pocket can cause disease.

## Methods

### Modeling and simulations

Models of wild-type and mutant human Cx50 hemichannels were built using the MODELLER software [56] and the sheep Cx50 intercellular channel structure as template, which is in an open conformation (PDB ID: 7JJP; [7]). The missing intracellular loop in the template structure (amino acid residues 97 to 144) was not modeled and the resulting N- and C-terminal residues from this gap were neutralized. The C-terminal domain (amino acid residues 228–433) was not modeled because of the lack of template. The total amount of Cx50 residues modeled was 178. The simulation protocol included several steps to minimize the possible influence an open conformation template might have upon determination of the final structure. One thousand models were generated with MODELLER for each system, and the best model was selected using MAIDEN software, which uses an energy function designed exclusively for membrane proteins [57]. The best models of Cx50 hemichannels were placed in a palmitoyl-oleoyl-phosphatidylcholine (POPC) bilayer of 150 Å × 150 Å × 135 Å dimensions using the online software CHARMM-GUI [58]. Water molecules (TIP3 model) and 0.1 M KCl were added to the system. The topologies were produced using the CHARMM v32 forcefield [59]. Energy minimizations were run using the GROMACS (v. 2019) software [60] and the steepest descent algorithm. A series of short molecular dynamics simulations were run at NVT ensemble starting with a position restraint in all non-hydrogen atoms, after which these restraints were gradually removed. The temperature was held at 310ºK using a Berendsen thermostat. Then, equilibration molecular dynamics simulations were run for 50 ns using an NPT ensemble. From then on, the temperature was held at 310°K using a Nose–Hoover thermostat and pressure was held at 1 atm using a Parrinello–Rahman barostat. Afterwards, a second series of 50-ns equilibrations were run applying a 0.1 V/nm external electric field in the z-axis to simulate a membrane voltage difference. Finally, three production simulations of 100 ns were run in which the velocities of the initial frame were randomized from a Maxwell distribution, while temperature, pressure and external electric field were kept the same as in the 50-ns equilibration series. Positions were recorded every 1 ps resulting in trajectories of 100,000 steps. Analyses of simulations including distance measurements were performed using GROMACS packages and the MDAnalysis module of Python [61].

Movement of transmembrane domains was calculated as the distance between the C_α_ atoms from amino acid residues 25 and 159 (TM1–TM3 distance) and amino acid residues 85 and 220 (TM2–TM4 distance), because these residues are closest to the center of mass of each transmembrane domain. Inclination of the transmembrane domains was measured as the angle formed by the best straight line passing through the C_α_ atoms of all amino acid residues of TM1 and that of the C_α_ of all amino acid residues in each of the other transmembrane domains. The number of water molecules inside the intracellular pocket was calculated as defined by Araya-Secchi et al. [11], i.e., water molecules at a distance of ≤ 6.0 Å form the center of mass of the C_α_ of amino acid residues comprising the pocket (i.e., N9, E12, E13, E16, S18, R23, L26, T27, F30, R33, I34, Q81, V85, S86, P88, S89, Y92, V93, L148, Y151, H154, I155, K158, T159, E162, S216, L219, N220, E223, L227).

### Generation of human Cx50 constructs

Cx50 constructs were generated by PCR using High Fidelity Phusion DNA polymerase (New England BioLabs, Ipswich, MA, USA). The different mutants were obtained using the coding region of the wild-type human *GJA8* gene subcloned into pSFFVneo or pBSSK-Xg as the template [62]. Primers were designed in opposite directions to incorporate the desired mutations into the PCR product: for Cx50R33E, GAGATCCTCATCCTTGGCACGGCC (sense) and GAAGATGAAAAGCACGGTGAGCCAGAC (antisense); for Cx50E162R, AGTGGGCTTCATCGTGGGCCACTAC (sense) and CGAAAGAGGGTCTTGAAGATGATGTGGCAG (antisense). To obtain the double mutant (Cx50R33E,E162R), we used Cx50R33E in pSFFV-neo and Cx50R33E in pBSSK-Xg as templates and the set of primers of Cx50E162R.

All constructs were fully sequenced at the Cancer Research Center DNA Sequencing Facility of the University of Chicago to verify that PCR amplification did not introduce random mutations.

### Cell culture

HeLa cells were grown in MEM supplemented with non-essential amino acids, 10% fetal bovine serum, 2 mM glutamine, 100 units/mL penicillin G and 10 μg/mL streptomycin sulfate. To obtain stably transfected cells, cells at ~ 50% confluence were transfected with wild-type or mutant Cx50 in pSFFV-neo using Opti-MEM, Lipofectin and PLUS Reagent (ThermoFisher Scientific, Waltham, MA, USA); clones were selected by their resistance to 1 mg/mL geneticin (ThermoFisher Scientific) as previously performed [62].

### Immunofluorescence

Cells were grown on glass coverslips until they reached ~ 80% confluence. Then, they were fixed in 4% paraformaldehyde in phosphate buffered saline pH 7.4 (PBS) for 15 min and subjected to immunofluorescence using previously characterized rabbit polyclonal anti-Cx50 antibodies [62] and Cy3-conjugated goat anti-rabbit IgG antibodies (Jackson ImmunoResearch, West Grove, PA, USA). The cellular distribution of Cx50 was studied with a Zeiss Plan Apochromat 40× objective (n.a., 1.0) in an Axioplan 2 microscope (Carl Zeiss, Munich, Germany) equipped with a mercury lamp. Images were acquired with a Zeiss AxioCam digital camera using Zeiss AxioVision software, keeping light intensity and exposure time constant for all images within an experiment. Figures were generated in Adobe Photoshop 23.5.5 (Adobe Systems, Inc., San Jose, CA, USA).

### Intercellular communication

Parental (untransfected) HeLa cells and HeLa cells stably transfected with wild-type Cx50, Cx50R33E, Cx50E162R or Cx50R33E,E162R were grown on glass coverslips until they reached 90‒95% confluence. Then, one cell within a cluster was microinjected with a solution containing 9% Neurobiotin (charge: + 1; MW: 287.2; Vector Laboratories, Burlingame, CA, USA) and 5% Lucifer yellow (charge − 2; MW: 444.4; Sigma-Aldrich, St. Louis, MO, USA) for 1 min using a picospritzer (model PLI-188; Nikon Instruments Inc., Melville, NY, USA) [63]. After allowing the microinjected gap junction tracers to diffuse to neighboring cells for 10 min, cells were fixed in 4% paraformaldehyde for 15 min and incubated with Cy3-streptavidin conjugate (Sigma-Aldrich) to detect Neurobiotin by fluorescence microscopy [63]. Lucifer yellow facilitated identification of the injected cell, because human Cx50 has limited permeability to this dye and did not spread [64]. The extent of Neurobiotin intercellular transfer was determined by counting the number of adjacent cells containing the tracer. The number of microinjections ranged from 10 to 24 for cells expressing the different constructs. Data are presented as mean ± S.E.M. Statistical analysis was performed using Student’s t-test. Graphs were generated in SigmaPlot 10 (Systat Software, Inc., Palo Alto, CA, USA).

### Connexin hemichannel activity

*Electrophysiological studies.* Female *Xenopus laevis* were purchased from Nasco (Nasco Education, Fort Atkinson, WI, USA) and maintained in the animal facility of the Facultad de Ciencias, Universidad de Valparaíso. Females were anesthetized with tricaine and subjected to a partial ovariectomy. *Xenopus* oocytes were treated with collagenase type II (Worthington Biochemical Corp, NJ, US) and defolliculated. Four hours after defolliculation, oocytes were microinjected with 41 nL of 1 µg/µL antisense Cx38 oligonucleotide (GCTTTAGTAATTCCCATCCTGCCATGTTTC) (Integrated DNA Technologies, Coralville, IA, USA) to efficiently reduce endogenous expression of Cx38 [65] using a Drummond Scientific Nanoinjector (Broomall, PA, USA). At the same time or a few hours later, oocytes were microinjected with 41 nL of a solution containing 1 µg/µL wild-type or mutant Cx50 cRNA. Oocytes were maintained at 18°C in ND-96 medium (96 mM NaCl, 2 mM KCl, 1.8 mM CaCl_2_, 1 mM MgCl_2_, 5 mM HEPES, pH 7.5 adjusted with NaOH).

Macroscopic hemichannel currents were evaluated 24‒72 h post-cRNA injection in oocytes that had been previously microinjected with 30 nL of 5 µM BAPTA (1,2-Bis(2-aminophenoxy)ethane-*N*,*N*,*N*′,*N*′-tetraacetic acid) to block activation of endogenous Ca^2+^-activated chloride channels, and allowed to recover in ND-96 medium for 30 min. All experiments were performed at room temperature (20–22°C) in an extracellular solution containing 1.8 mM Ca^2+^. Macroscopic hemichannel currents were recorded using the two-electrode voltage-clamp technique using a Warner oocyte clamp (OC-725C; Warner Instruments, Holliston, MA, USA). Micropipettes housing the two Ag/AgCl electrodes (i.e., the voltage electrode used to measure intracellular potential and the electrode used to inject current into the oocyte) were pulled to a resistance of 0.5 to 1.5 MΩ and filled with 3 M KCl. The bath solution contained 118 mM NaCl, 2 mM KCl, 1.8 mM CaCl_2_ and 5 mM HEPES, pH 7.4. The bath was connected to two 3 M KCl pools using agar salt bridges. Oocytes under two-electrode voltage clamp were depolarized from − 80 mV to + 40 mV from a holding potential of − 80 mV in 20-mV increments and then returned to − 80 mV. The current vs. voltage relationships (I/V curves) were determined from the peak of the tail currents. Currents were low-pass filtered at 200 Hz and sampled and digitized at 2 kHz using an A/D converter (National Instruments, Austin, TX). Currents were analyzed using pClamp 10.4 (Axon Instruments, San Jose, CA). Graphs were generated in GraphPad Prism 6.0 (GraphPad Software, Inc., San Diego, CA, USA).

#### Uptake of a connexon-permeant tracer

Dye uptake was performed on HeLa cells as previously described [26]. Briefly, parental HeLa cells or HeLa cells stably transfected with wild-type Cx50, Cx50R33E, Cx50E162R or Cx50R33E,E162R were grown on 25-mm diameter coverslips for 24 h or until they reached 50–70% confluence. Then, the culture medium was removed and replaced by Hanks’ balanced salt solution containing divalent cations (140 mM NaCl, 5.3 mM KCl, 0.1% glucose, 0.34 mM Na_2_HPO_4_, 1.26 mM CaCl_2_, 0.5 mM MgCl_2_, 10 mM HEPES, pH 7.4) and 5 μM ethidium bromide. After 20 min, the media was replaced by a divalent cation-free (DCF) solution containing 5 μM ethidium bromide. Twenty minutes later, the solution was changed to DCF solution containing 5 μM ethidium bromide and 100 μM La^3+^, a connexin hemichannel blocker. Fluorescence images were acquired using an Eclipse Ti-E inverted microscope equipped with a 40× objective (Nikon, Tokyo, Japan), a cooled digital camera (ORCA-FLASH 2.0; Hamamatsu Photonics, Shizuoka, Japan) and NIS-Element Advanced Research 4.3 software (Nikon). Time-lapse images were acquired in stream mode at 200 ms intervals at room temperature starting when the culture medium was replaced with Hanks’ balanced salt solution containing divalent cations. Data are shown as F/F_0_ ratio (arbitrary units; a.u), where F and F_0_ are the background-subtracted fluorescence intensities at the beginning of the recording (F_0_) and at each time point of recording afterwards (F). The data points were fitted to a linear equation (y = ax + b) using Microsoft Excel. Changes in the slope (a) of the F/F_0_ curve were analyzed for significance using Microsoft Excel. Plots were generated using GraphPad Prism 6.0. Phase-contrast micrographs were captured at the beginning and at the end of the recording period to confirm that cells maintained their normal shape during the recording protocol, as a sign of their viability.

#### Measurement of ATP release

Untransfected HeLa cells or HeLa cells stably transfected with wild-type or mutant Cx50 were plated on 24-well plates at 10,000 cells per well. Twenty-four hours later, the incubation media was replaced by Hanks’ balanced salt solution containing divalent cations or by DCF solution. After 30 min, 5-μL samples of incubation medium were collected and the amount of ATP released was determined using the ATP Determination Kit (Invitrogen, Eugene, OR, USA), a luciferin-luciferase bioluminescence assay. The samples were mixed with 45 μL ATP-mix solution in a 96-well plate, and the luminescence was determined using an Appliskan luminometer (ThermoFisher Scientific). The concentration of extracellular ATP was normalized by the levels of wild-type or mutant Cx50 proteins determined in the corresponding well by chemiluminescent immunolabeling. After sample collection, cells were fixed with 4% paraformaldehyde in PBS followed by permeabilization with 1% Triton X-100 in PBS. Then, cells were incubated with blocking solution (1% Triton X-100, 2% bovine serum albumin in PBS) for 1 h, followed by rabbit polyclonal anti-Cx50 antibodies for 3 h. After several rinses with PBS, cells were incubated for 1 h with horseradish peroxidase-conjugated donkey anti-rabbit IgG antibodies (Jackson ImmunoResearch Laboratories Inc.; catalog number: 711-036-152) followed by several rinses in PBS. Binding of secondary antibody was determined by incubation with the Clarity Max Western ECL Substrate kit following the manufacturer's instructions (Bio-Rad, Hercules, California, USA; catalog number: 1705062). Detection of the chemiluminescence signal intensity was determined using an Appliskan luminometer (ThermoFisher Scientific).

### Statistical analysis

Statistical analysis was performed using the NumPy module from Python (for Molecular Dynamics data), Student’s t-test in Microsoft Excel (for intercellular communication, ethidium uptake and ATP release), GraphPad Prism 6.0 (for electrophysiological studies).

### Supplementary Information

**Additional file 1: Figure S1.** Temporal course of fluctuations during the simulations. Left panel, Graph shows the time-course of root mean square deviation (RMSD) for wild-type and mutant Cx50 hemichannels in a 100-ns simulation. Right panel, Graph shows the root mean square fluctuation (RMSF) around each amino acid residue (aa) for wild-type and mutant Cx50 hemichannels in a 100-ns simulation. The vertical shades mark the position of the amino acid residues belonging to the N-terminal helix (mauve) and the transmembrane domains (light mint green). Data are presented as mean (central line) ± standard deviation (shade) from three independent 100-ns simulations ran for wild-type Cx50 (green), Cx50R33E (light blue), Cx50E162R (orange), Cx50E162Q (yellow) and Cx50R33E,E162R (purple) hemichannels.

## Data Availability

All data generated or analyzed are included in the manuscript except the Molecular Dynamics data which will be made available upon request.

## Abbreviations

Cx: Connexin
DCF: Divalent cation-free
I_max_: Maximal current
PBS: Phosphate buffered saline
POPC: Palmitoyl-oleoyl-phosphatidylcholine
RMSD: Root mean square deviation
RMSF: Root mean square fluctuation
TM1: Transmembrane domain 1
TM2: Transmembrane domain 2
TM3: Transmembrane domain 3
TM4: Transmembrane domain 4
