# Supplementary material for: Connexin channels and hemichannels are modulated differently by charge reversal at residues forming the intracellular pocket
Source: Biol Res. 2024 May 23;57:31. doi: 10.1186/s40659-024-00501-5 (PMC11112876; doi:10.1186/s40659-024-00501-5)
Supplement: Supplementary file 1 — Additional file 1: Figure S1. Temporal course of fluctuations during the simulations. Left panel, Graph shows the time-course of root mean square deviation (RMSD) for wild-type and mutant Cx50 hemichannels in a 100-ns simulation. Right panel, Graph shows the root mean square fluctuation (RMSF) around each amino acid residue (aa) for wild-type and mutant Cx50 hemichannels in a 100-ns simulation. The vertical shades mark the position of the amino acid residues belonging to the N-terminal helix (mauve) and the transmembrane domains (light mint green). Data are presented as mean (central line) ± standard deviation (shade) from three independent 100-ns simulations ran for wild-type Cx50 (green), Cx50R33E (light blue), Cx50E162R (orange), Cx50E162Q (yellow) and Cx50R33E,E162R (purple) hemichannels. [file 40659_2024_501_MOESM1_ESM.pdf]

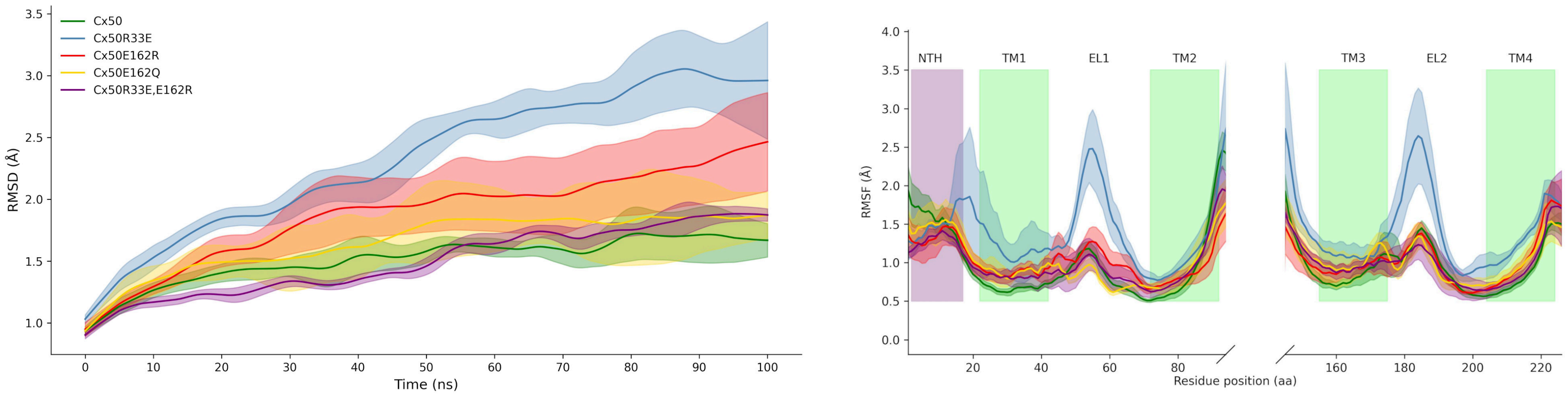

Figure S1 Temporal course of fluctuations during the simulations. Left panel, Graph shows the time-course of root mean square deviation (RMSD) for wild-type and mutant Cx50 hemichannels in a 100-ns simulation. Right panel, Graph shows the root mean square fluctuation (RMSF) around each amino acid residue (aa) for wild-type and mutant Cx50 hemichannels in a 100-ns simulation. The vertical shades mark the position of the amino acid residues belonging to the N-terminal helix (mauve) and the transmembrane domains (light mint green). Data are presented as mean (central line) ± standard deviation (shade) from three independent 100-ns simulations ran for wild-type Cx50 (green), Cx50R33E (light blue), Cx50E162R (orange), Cx50E162Q (yellow) and Cx50R33E,E162R (purple) hemichannels
